# Ashwagandha Extract as a Promising Source of Immunology Support for *Apis mellifera*

**DOI:** 10.3390/ani16152328

**Published:** 2026-07-30

**Authors:** Patrycja Staniszewska, Anna Gryboś, Adam Staniszewski, Agata Kiec, Magdalena Podlodowska, Krzysztof Olszewski, Aneta Strachecka

**Affiliations:** 1 Department of Invertebrate Ecophysiology and Experimental Biology, Faculty of Environmental Biology, University of Life Sciences in Lublin, 20-950 Lublin, Poland; 2 Subdepartment of Apidology, Institute of Biological Basis of Animal Production, Faculty of Animal Sciences and Bioeconomy, University of Life Sciences in Lublin, 20-950 Lublin, Poland

**Keywords:** *Apis mellifera*, supplementation, *Withania somnifera*, proteolytic system

## Abstract

In response to the growing problem of immune decline in honeybees (*Apis mellifera*), we investigated the potential of Ashwagandha (*Withania somnifera*) extract as a natural dietary supplement. The experiment was conducted under constant laboratory conditions, and worker bees were divided into three groups: a control group fed standard sugar syrup without additives, experimental group A1 receiving syrup supplemented with a low concentration of Ashwagandha extract, and experimental group A2 receiving a higher concentration of the extract. Hemolymph samples were analyzed at regular intervals for total protein concentration, protease and protease inhibitor activities, as well as non-enzymatic markers such as glucose and triglycerides. Ashwagandha supplementation increased total protein concentration and stimulated the proteolytic system in both experimental groups, with generally stronger effects in bees receiving the higher dose. At the same time, the balance between proteases and their inhibitors was maintained, indicating a coordinated and controlled physiological response. The supplemented bees also showed higher levels of glucose and triglycerides, suggesting improved energy status and metabolic support. Overall, the results indicate that Ashwagandha extract may act as a promising natural supplement supporting honeybee physiological resistance and immune homeostasis.

## Simple Summary

The objective of this research was to investigate how a diet supplemented with Ashwagandha extract influences the immune resistance of the honey bee (*Apis mellifera*). Honey bee workers were divided into a control group (fed glucose syrup) and two experimental groups (fed glucose syrup with a low or high dose of the extract). Hemolymph was sampled across five age intervals (days 1, 7, 14, 21, and 28) to measure basic metabolic components (glucose and triglycerides) and proteolytic system parameters (proteases, protease inhibitors, and total protein), which are crucial for pathogen defense. Dietary administration of Ashwagandha led to a significant increase in total protein and overall biomarker concentrations. Notably, the stimulation of the proteolytic system was more robust in the group receiving the higher dose. Conclusively, these data suggest that Ashwagandha extract successfully boosts the innate immunity of honey bees.

## 1. Introduction

The anthropogenic pressure on the natural environment resulting from the intensification of agriculture, urbanization, and industrial development leads to progressive environmental degradation and loss of biodiversity, significantly disrupting the functioning of ecosystems [1]. One of the most vulnerable groups of organisms is pollinating insects, including the honeybee (*Apis mellifera*), a key species responsible for pollination, cross-fertilization and, as a result, maintaining the biodiversity of entomophilous plants [2,3]. Bee health directly impacts the stability of agricultural production, food security, and the economies of many countries. Unfortunately, recent decades have seen a rapid decline of the bee population and an increasing rate of their disappearance, the etiology of which is multifactorial and includes the effects of pathogens, parasites, environmental toxins, nutritional deficiencies, oxidative stress, and climate change [4]. The consequence of the reduced immunity of bees is an even greater susceptibility of the colony to stress and disease factors (often secondary infections) [5,6,7,8]. Therefore, there is growing interest in natural substances with potential immunomodulatory effects that could support the functioning of bee colonies.

**Scheme 1:**
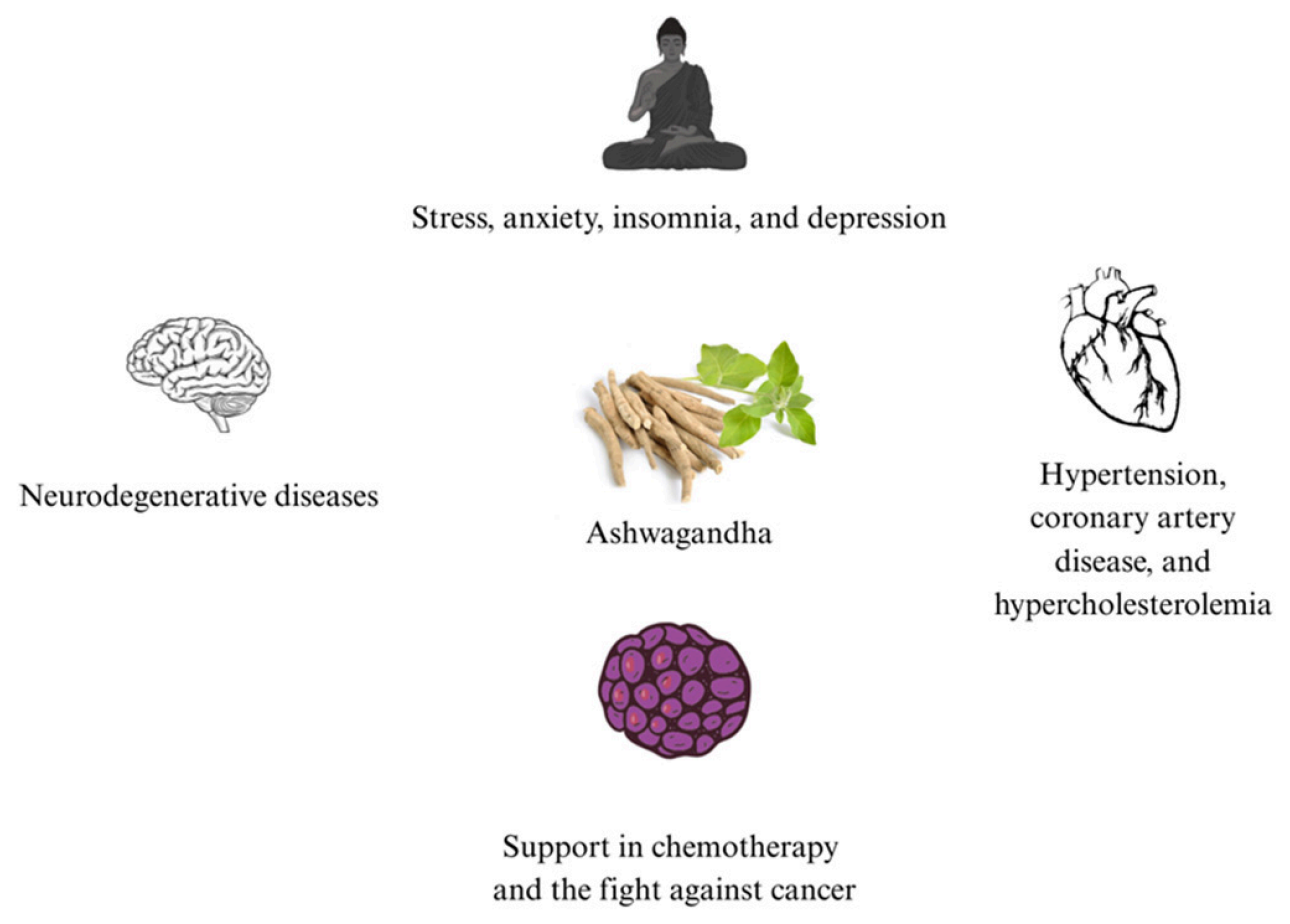
Use of Ashwagandha as support in medicine or therapy.

One of the most promising plant raw materials is *Withania somnifera*, known as Ashwagandha, which has been used in Ayurvedic medicine for centuries due to its adaptogenic, anti-inflammatory, antioxidant, and immunomodulatory properties (Scheme 1) [9,10]. *W. somnifera* extracts contain a number of bioactive compounds, primarily withanolides, of which withaferin A is one of the best known [11]. In vitro studies on B-cell tumor cell lines have demonstrated the ability of withaferin A to induce potent cytotoxicity, induce apoptosis, inhibit chaperone proteins (HSP90), and disrupt cell survival signals [12]. Studies conducted in humans and animal models have shown that extracts from this plant can increase immunoglobulin levels, the activity of T and B lymphocytes and NK cells, and the production of cytokines such as IFN-γ. At the molecular level, withaferin A has been documented to regulate the Bcl-2/Bax ratio, activate caspases, and inhibit the transcription factor NF-κB, which is associated with anticancer and anti-inflammatory effects [13,14]. Moreover, the literature describes the synergistic effect of withaferin A with CAPE (caffeic acid phenethyl ester), a propolis component with strong immunomodulatory and antioxidant properties [15]. The use of nanoparticle formulations of Ashwagandha extract further increases their bioavailability and biological activities by modulating the PI3K/Akt and MAPK signaling pathways [16,17].

The key mechanisms of anti-infective immunity in bees are antioxidant and proteolytic, which are closely interconnected. Both the immune proteins of the antioxidant and proteolytic systems are synthesized in a tissue called the fat body. Then, the insect equivalent of blood—hemolymph—washes the fat body and distributes the produced compounds throughout the honeybee’s body. Therefore, hemolymph is used in addition to the fat body when testing the activity of the proteolytic system components. The proteolytic system consists of proteases and their inhibitors. Their main task is the proteolysis of foreign proteins, e.g., pathogens. In addition, specific proteases are involved in immune pathways, such as the melanization process. Proteases and their inhibitors can be divided according to the types of compounds found in their active center and the pH at which they are active (acidic, alkaline, and neutral). Proteases include the most numerous serine proteases. In addition, we distinguish between metalloproteases, cysteine proteases, and aspartic proteases. Maintaining a balance between proteases and their inhibitors is crucial for the bee’s immunity. Moreover, the activity of the proteolytic system depends on the caste, season, and, importantly, the level of environmental pollution. For example, when exposed to plant protection products, including pesticides, the activity of this system decreases [18,19,20,21].

There are also other vital parameters related to the functioning of the honeybee organism that are particularly significant from the perspective of dietary supplementation. Glucose, as a non-enzymatic marker of carbohydrate metabolism, serves as a direct fuel for processes such as flight, thermoregulation, and royal jelly secretion, derived from nectar or supplements (sucrose syrup, invert syrup, and corn syrup) following hydrolysis by invertase. In the hemolymph, it coexists with trehalose—the primary circulating sugar—forming energy biomarkers whose levels reflect metabolic intensity, colony health, and resistance to stressors (starvation, rain, wintering). Their concentrations fluctuate under the influence of environmental factors, with a strong but complex correlation. Additionally, triglycerides (TAG)—the primary simple lipids (90% of the lipid pool)—serve as markers of energy storage and mobilization, synthesized from carbohydrates in the fat body, from where they are transported as diglycerides (DAG) in the hemolymph. Derived mainly from pollen (0.94–24.6% depending on botanical origin [22]), TAGs reflect nutritional balance, energy requirements, and oxidative stress; their fluctuations are evident under stressors, e.g., *Varroa destructor* infection or glucose deficiency, which can trigger lipolysis of reserves. Fat, analogous to the vertebrate liver and hormonally regulated (adipokinetic hormone, octopamine), varies with age, caste, and conditions (e.g., higher mass in winter bees). Together, glucose and TAGs monitor energy homeostasis: glucose reflects current consumption, while TAGs indicate reserves and stress; abnormalities in these markers signal metabolic dysfunction in monocultures or under the influence of pathogens or pesticides. Understanding these markers is crucial for ecophysiology and beekeeping [22,23,24]. *W. somnifera* shows contrasting effects depending on the organism: in mammals, it is known as an adaptogen that supports homeostasis and immune function, whereas in some insect pests, such as *Spodoptera litura* and *Chrysomya megacephala*, it acts as a toxic agent that disrupts development and metamorphosis, likely through interference with ecdysone signaling. This duality justifies the selection of the *A. mellifera* extract for supplementation in controlled, adjusted syrup doses, enabling the evaluation of its potential support of humoral innate immunity through modulation of protease and inhibitor activities, without the risk of toxicity observed in phytophages.

Given the growing threats associated with negative human impact and the need to seek new methods for pollinator protection, analyzing the immunomodulatory potential of *W. somnifera* in *A. mellifera* is both scientifically justified and relevant to beekeeping practice. The aim of this article is to enrich the general body of knowledge on the properties of *W. somnifera* extracts and to assess their potential use as a natural agent supporting the honeybee proteolytic system.

## 2. Materials and Methods

### 2.1. The Cage Part of the Experiment

The study used 1-day-old worker bees (*A. mellifera carnica*) sourced from the University of Life Sciences in Lublin (coordinates: 51°13′31″ N, 22°38′07″ E). Preparation of one-day-old worker bees followed the protocol outlined by Strachecka et al. [25]. Fifty bees were placed in each of 30 wooden cages with dimensions of 12 × 12 × 4 cm (length × height × width) with a total volume of 576 cm^3^. Each cage was equipped with a sliding front window, side ventilation openings, and a feeder positioned on the top, consisting of a syringe fitted with a modified adapter. The experimental environment was maintained in an air-conditioned chamber at a constant temperature of 35 °C and 65% relative humidity to ensure optimal conditions.

The cages were divided into the following groups (10 cages per group):

(1) Control group (C) where bees were fed with glucose syrup (1 L H_2_O: 1 kg of sugar),
(2) Experimental group (A1) where bees were fed with glucose syrup (prepared as in the control group) with the addition of Ashwagandha extract in the proportion: 500 mL syrup + 10 µL extract,
(3) Experimental group (A2) where bees were fed with glucose syrup (prepared as in the control group) with the addition of Ashwagandha extract in the proportion: 500 mL syrup + 20 µL extract.

The ashwagandha extract used in this experiment was purchased from a certified company, Mother’s Protect, Flower One Sp. z o.o., Chwaszczyno, Poland. The bees in cages were fed ad libitum, and the syrup remaining in the syringes was replaced every 2 days. The appropriate type of supplementation (appropriate dosage) was given to the groups throughout the experiment.

### 2.2. Analytical Part

Hemolymph sampling was scheduled at 7-day intervals, with the first sampling on day 1 of the experiment. Hemolymph was collected from three bees per cage in each group (1 collection = 3 groups × 18 bees).

**Scheme 2:**
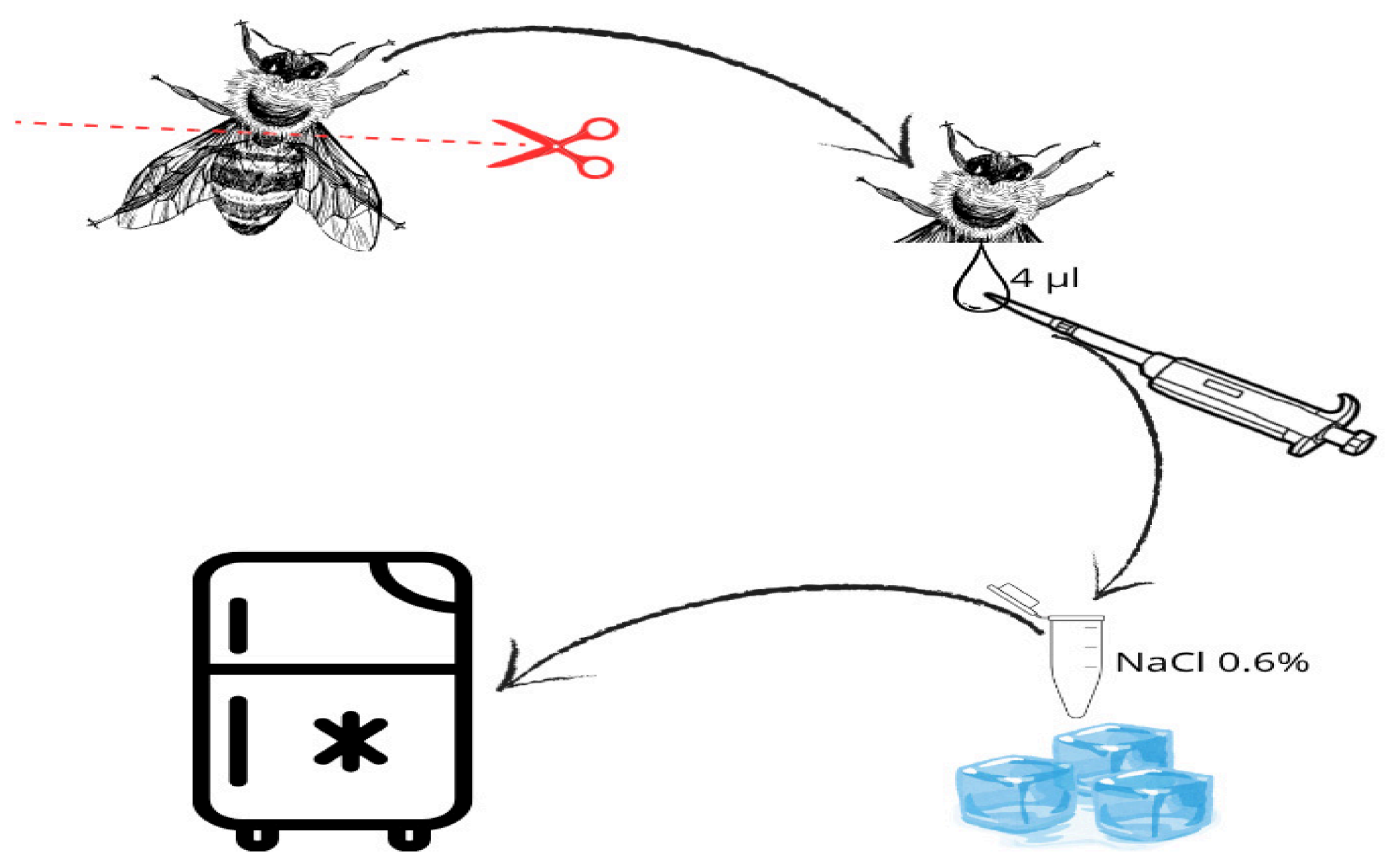
Method of collecting bee hemolymph.

To obtain hemolymph, the bee was held with tweezers, and a cut was made along the stylus. The incision was made using dissecting scissors. As a result of the incision and gentle pressure on the upper part of the bee’s body, a drop of hemolymph appeared at the incision site. It was collected using a pipette with a set volume of 4 μL placed in an Eppendorf tube containing 200 µL of ice-cooled 0.6% NaCl. The samples were immediately refrigerated at −25 °C for further biochemical analyses (Scheme 2).

The following biochemical analyses were performed in the hemolymph from individual samples:

(1) Total protein concentration was determined using the Lowry method, as modified by Schacterle and Pollack [26].
(2) Proteolytic system activity was determined as follows:
  - activities of acidic, neutral, and alkaline proteases according to the Anson method modified by Strachecka and Łoś [27];
  - activities of acidic, neutral, and alkaline protease inhibitors according to the Lee and Lin method [28];
(3) Non-enzymatic markers were determined using a commercial kit (TRÓJGLICERYDY DST Alpha Diagnostic kit; GLUKOZA OXY DST Alpha Diagnostic kit, Alpha Diagnostic International, San Antonio, TX, USA) with modified instructions by Łoś and Strachecka [27].

### 2.3. Statistical Analysis

Data analysis was conducted using SAS OnDemand for Academics (SAS Institute Inc., Cary, NC, USA), running on SAS version 9.4 (Maintenance Level 8). A two-factor ANOVA (General Linear Model, GLM procedure) was performed with two independent variables: the group category (C, A1, A2) and the age of the bees (1, 7, 14, 21, and 28 days old). The test’s power, which indicates the probability of detecting a true effect, was assessed using the GLMPOWER procedure and found to exceed 80%, ensuring the test’s reliability. The normality of the distribution was verified within experimental groups using the Shapiro–Wilk test, and homogeneity of variances was tested using Levene’s test. Post-hoc multiple comparisons were performed using the Spjøtvoll-Stoline (Unequal N HSD) test.

## 3. Results

### 3.1. Total Protein Concentration

**Figure 1:**
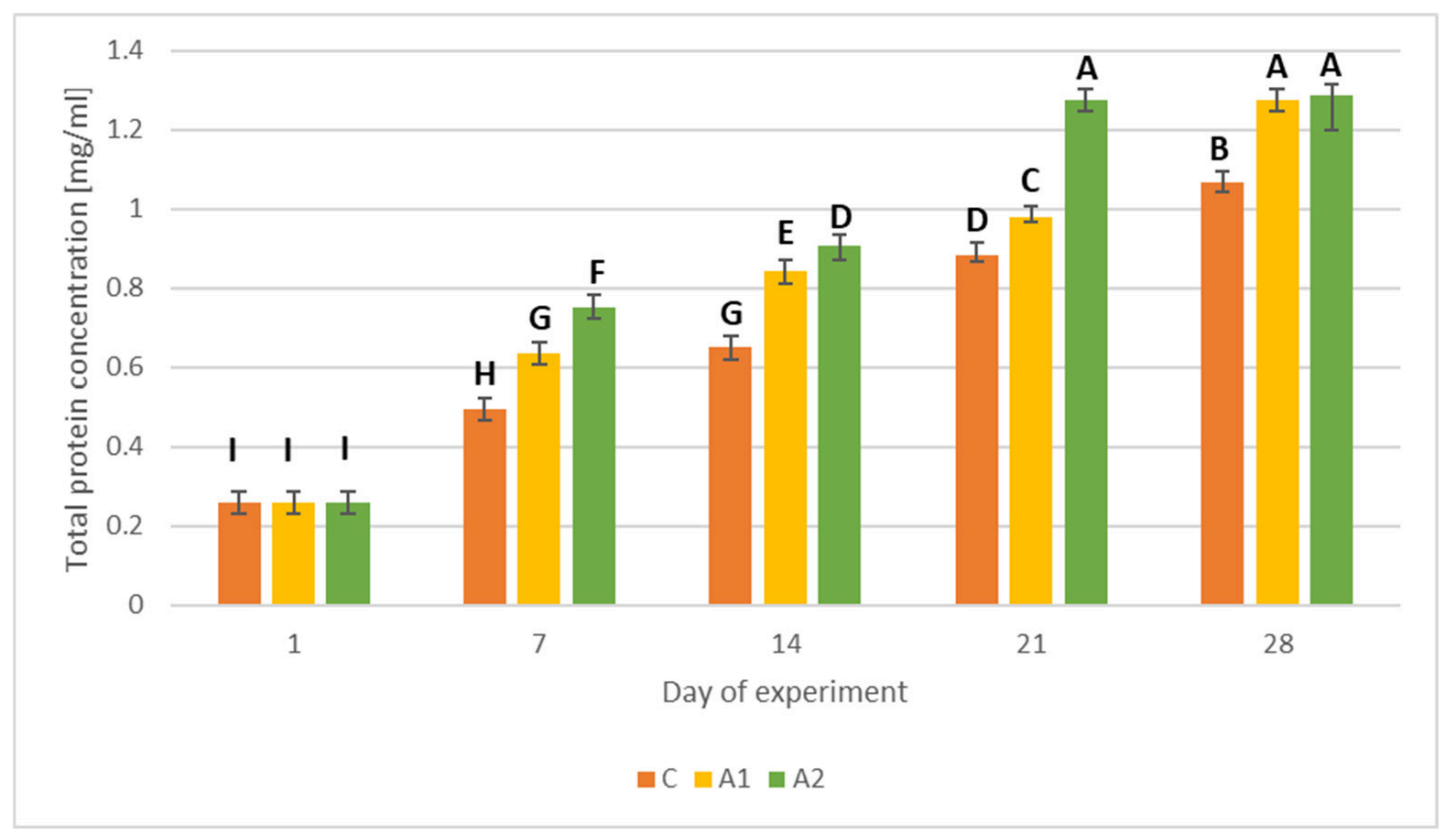
Total protein concentrations (bars represent means—$\overset{-}{x}$) in the hemolymph of workers in the control group (C)—not supplemented with Ashwagandha, and experimental groups A1, A2—supplemented with Ashwagandha (A1—10 µL of extract/500 µL of syrup, A2—20 µL of extract/500 µL of syrup). Two-Way ANOVA: supplementation method F(2, 222) = 423.24; *p* < 0.0001, se ± 0.003537–0.004248; days of supplementation: F(4, 222) = 1997.58, *p* < 0.0001, se ± 0.010557–0.018286; supplementation method × days of supplementation F(6, 222) = 11.89, *p* < 0.0001, se ± 0.004605–0.008115. Data are presented as least squares means (LS-means), and error bars represent standard deviations (SD). Different uppercase letters indicate statistically significant differences between groups (*p* < 0.05).

The total protein concentrations increased with the age of workers in all groups (Figure 1). In the groups of bees supplemented with Ashwagandha (A1 and A2), protein concentrations were always higher compared to the control group (C), with higher values observed in group A2 in bees between 7 and 21 days of age.

### 3.2. Acidic Protease Activities

The acidic protease activities increased with the age of workers. The highest acidic protease activities were observed on the 28th day of the experiment in all groups, and in bees supplemented with Ashwagandha, they were higher than in the control group. The largest increase was observed between days 14 and 21 of the experiment.

**Figure 2:**
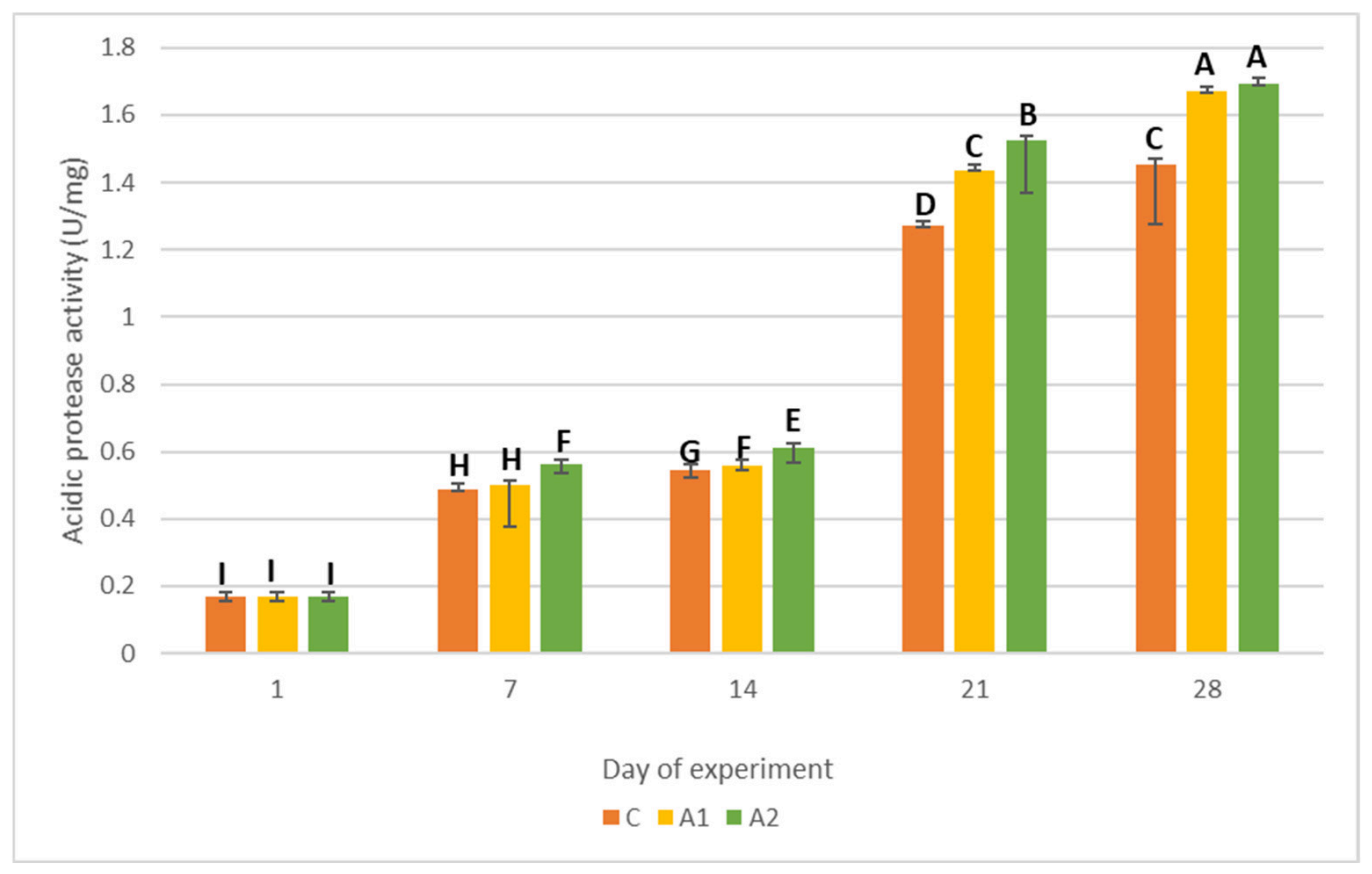
Acidic protease activities (bars represent means—$\overset{-}{x}$) in the hemolymph of workers in the control group (C)—not supplemented with Ashwagandha, and in experimental groups—supplemented with Ashwagandha (A1—10 µL of extract/500 µL of syrup, A2—20 µL of extract/500 µL of syrup). Two-Way ANOVA: supplementation method F(2, 222) = 73.02; *p* < 0.0001, se ± 0.00797072032–0.00957252546; days of supplementation: F(4, 222) = 2486.54, *p* < 0.0001, se ± 0.010557477–0.0158362155; supplementation method × days of supplementation F(6, 222) = 10.93, *p* < 0.0001, se ± 0.0158362155–0.0215171797. Data are presented as least squares means (LS-means), and error bars represent standard deviations (SD). Different uppercase letters indicate statistically significant differences between groups (*p* < 0.05).

### 3.3. Neutral Protease Activities

The neutral protease activities increased with the age of workers. The highest acidic protease activities were achieved on the 21st day of the experiment in experimental group A2 and on the 28th day of the experiment in experimental group A1. The largest increase was observed between the 14th and 21st day of the experiment in group A2. 

**Figure 3:**
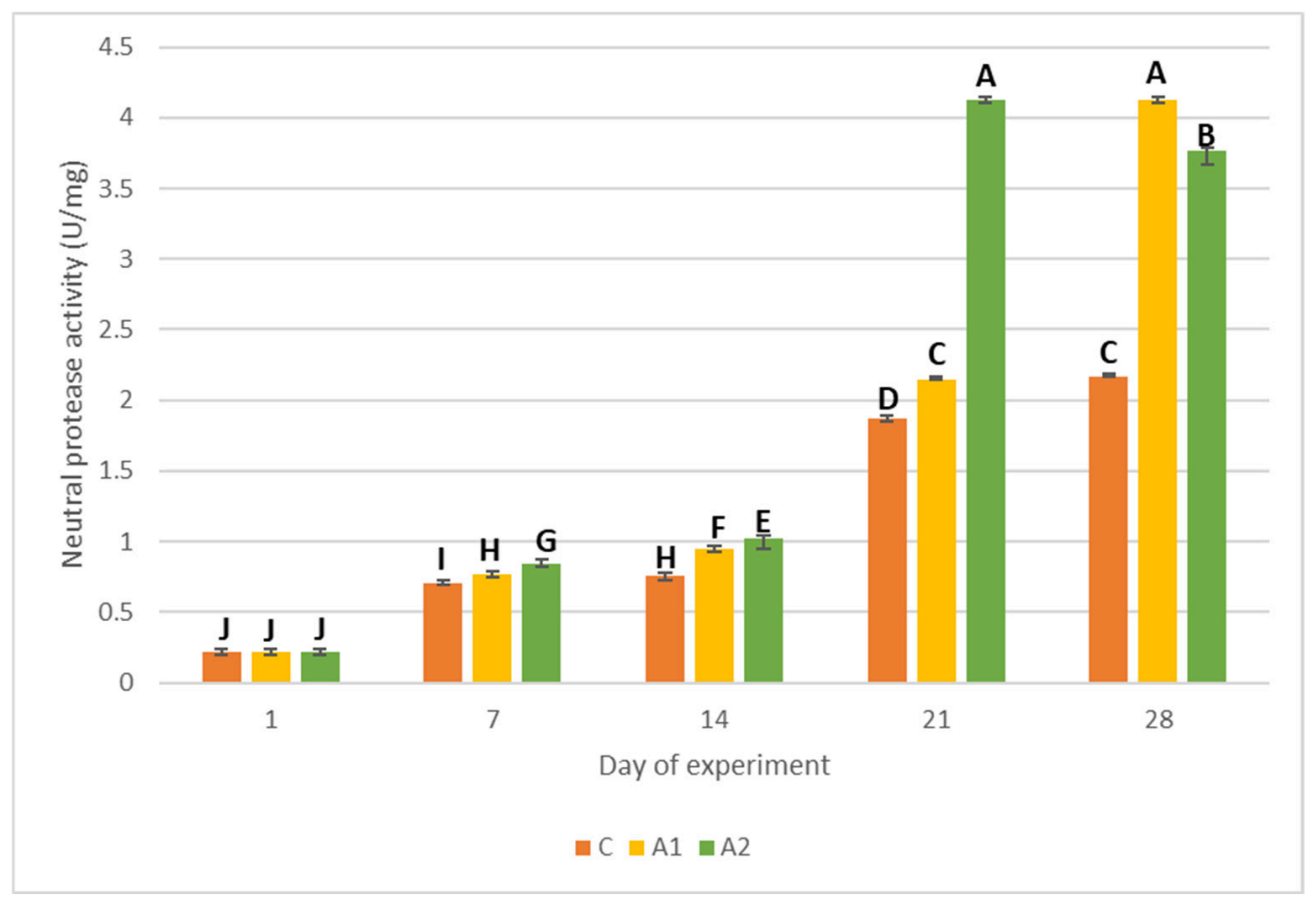
Neutral protease activities (bars represent means—$\overset{-}{x}$) in the hemolymph of workers in the control group (C)—not supplemented with Ashwagandha, and in experimental groups—supplemented with Ashwagandha (A1—10 µL of extract/500 µL of syrup, A2—20 µL of extract/500 µL of syrup). Two-Way ANOVA: supplementation method F(2, 222) = 15,960.8; *p* < 0.0001, se ± 0.00361631167–0.00434304984; days of supplementation: F(4, 222) = 56,902.5, *p* < 0.0001, se ± 0.00478992182–0.00718488274; supplementation method × days of supplementation F(6, 222) = 7141.49, *p* < 0.0001, se ± 0.00718488274–0.00976233326. Data are presented as least squares means (LS-means), and error bars represent standard deviations (SD). Different uppercase letters indicate statistically significant differences between groups (*p* < 0.05).

### 3.4. Alkaline Protease Activities

Alkaline protease activities increased with age until day 21 (in the case of group A2) or day 28 (in the case of groups C and A1). Protease activities were always higher in the hemolymph of bees supplemented with Ashwagandha compared to those not supplemented. In group A2, higher values were observed until day 21 of life. 

**Figure 4:**
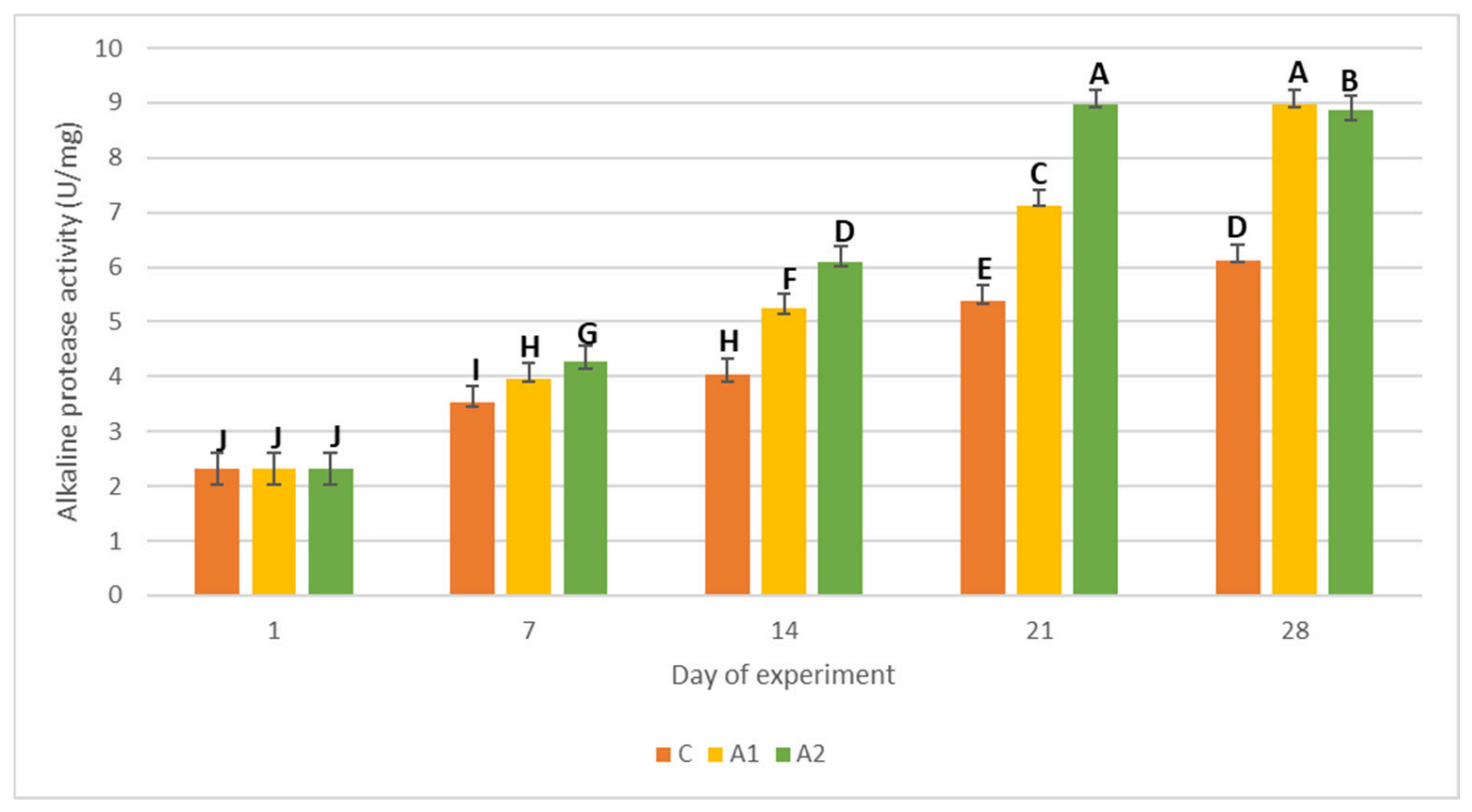
Alkaline protease activities (bars represent means—$\overset{-}{x}$) in the hemolymph of workers in the control group (C)—not supplemented with Ashwagandha, and in experimental groups–supplemented with Ashwagandha (A1—10 µL of extract/500 µL of syrup, A2—20 µL of extract/500 µL of syrup). Two-Way ANOVA: supplementation method F(2, 222) = 6114.27; *p* < 0.0001, se ± 0.0127929092–0.015363787; days of supplementation: F(4, 222) = 10,337.0, *p* < 0.0001, se ± 0.0169446222–0.0254169333; supplementation method × days of supplementation F(6, 222) = 613.58, *p* < 0.0001, se ± 0.0254169333–0.0345348118. Data are presented as least squares means (LS-means), and error bars represent standard deviations (SD). Different uppercase letters indicate statistically significant differences between groups (*p* < 0.05).

### 3.5. Acidic Protease Inhibitor Activities

The activities of acidic protease inhibitors in group A2 increased between days 1 and 7. Between days 7 and 14, a major decrease, only to be repaired in day 21 and an increase in day 28. In the control group, the tendency to increase remained steady throughout the experiment, while in group A1, the decrease occurred between days 21 and 28 of the experiment. The highest activities of acidic protease inhibitors occurred on the 28th day in group A2.

**Figure 5:**
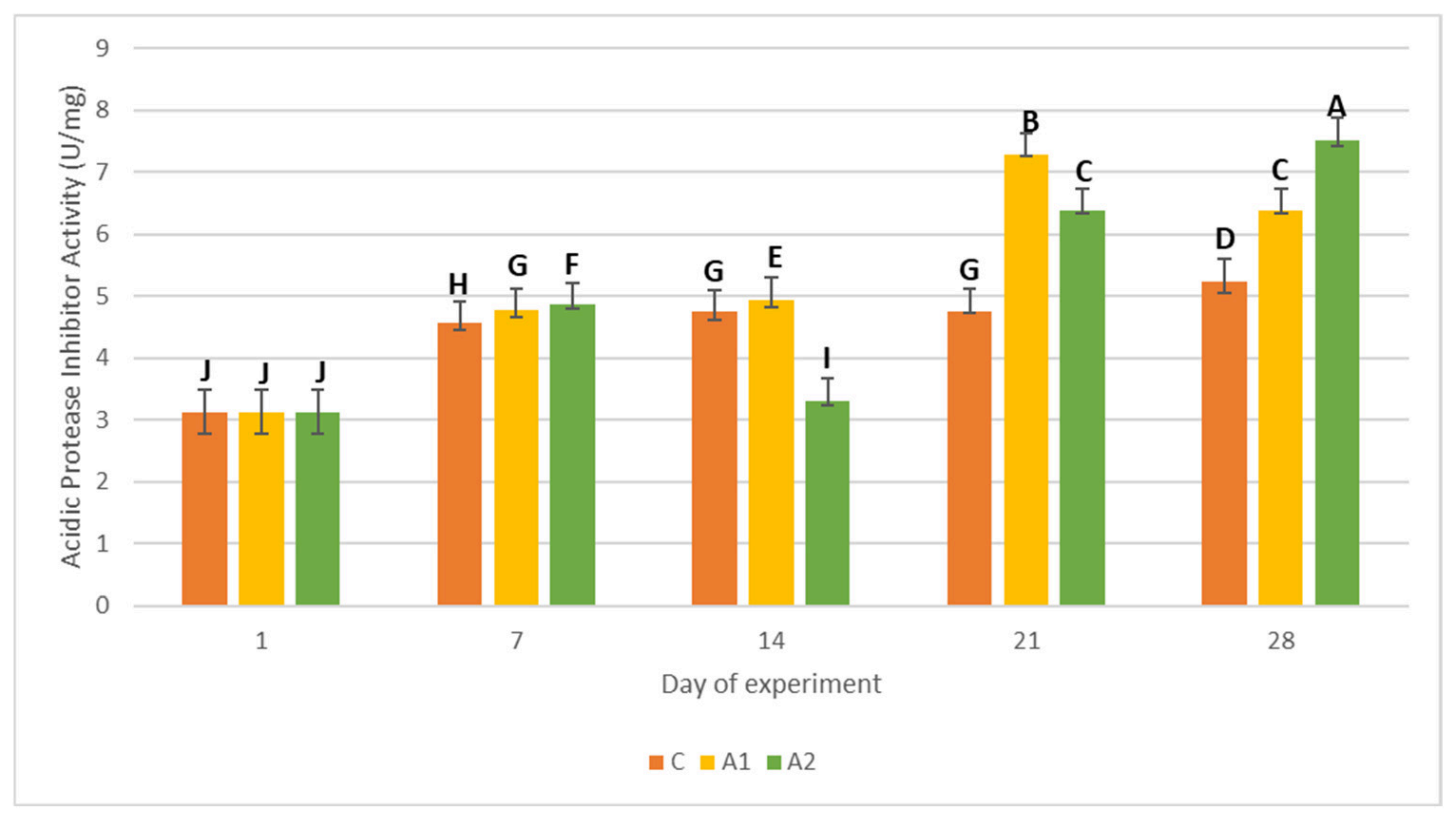
Acidic protease inhibitor activities (bars represent means—$\overset{-}{x}$) in the hemolymph of workers in the control group (C)—not supplemented with Ashwagandha, and in experimental groups—supplemented with Ashwagandha (A1—10 µL of extract/500 µL of syrup, A2—20 µL of extract/500 µL of syrup). Two-Way ANOVA: supplementation method F(2, 222) = 844.21; *p* < 0.0001, se ± 0.0154592905–0.0185660073; days of supplementation: F(4, 222) = 2352.00, *p* < 0.0001, se ± 0.0204763305–0.0307144957; supplementation method × days of supplementation F(6, 222) = 664.64, *p* < 0.0001, se ± 0.0307144957–0.0417327817. Data are presented as least squares means (LS-means), and error bars represent standard deviations (SD). Different uppercase letters indicate statistically significant differences between groups (*p* < 0.05).

### 3.6. Neutral Protease Inhibitor Activities

The activities of neutral protease inhibitors remained low from the 1st to the 14th day of the experiment. Between days 14 and 21, a major increase occurs in all groups of the experiment. Between days 21 and 28 in groups A1 and A2, a decrease in activity has been observed. The highest activities occurred on the 21st day in group A1 and on the 28th day in the control group.

**Figure 6:**
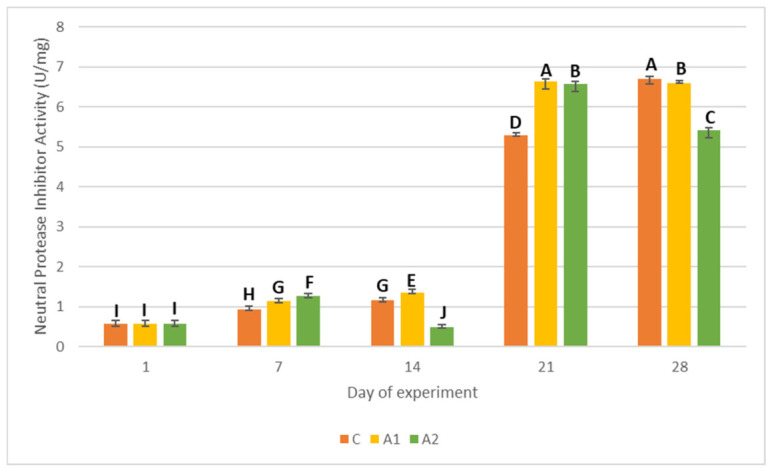
Neutral protease inhibitor activities (bars represent means—$\overset{-}{x}$) in the hemolymph of workers in the control group (C)—not supplemented with Ashwagandha, and in experimental groups—supplemented with Ashwagandha (A1—10 µL of extract/500 µL of syrup, A2—20 µL of extract/500 µL of syrup). Two-Way ANOVA: supplementation method F(2, 222) = 484.66; *p* < 0.0001, se ± 0.0104393823–0.0125372927; days of supplementation: F(4, 222) = 37,291.7, *p* < 0.0001, se ± 0.0138272997–0.0207409495; supplementation method × days of supplementation F(6, 222) = 567.91, *p* < 0.0001, se ± 0.0207409495–0.028181401. Data are presented as least squares means (LS-means), and error bars represent standard deviations (SD). Different uppercase letters indicate statistically significant differences between groups (*p* < 0.05).

### 3.7. Alkaline Protease Inhibitor Activities

Alkaline protease inhibitor activities increased in all groups until days 21 or 28 of worker life (except for a decrease in these activities in group A2 on day 14 of life). Activities were higher in groups supplemented with Ashwagandha (exception—A2 on day 14) compared to the control; these values were higher in group A1 than in A2 on days 14 and 21.

**Figure 7:**
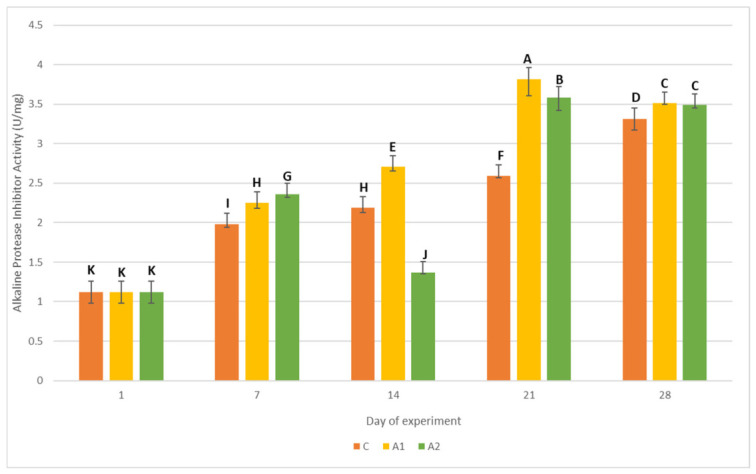
Alkaline protease inhibitor activities (bars represent means—$\overset{-}{x}$) in the hemolymph of workers in the control group (C)—not supplemented with Ashwagandha, and in experimental groups—supplemented with Ashwagandha (A1—10 µL of extract/500 µL of syrup, A2—20 µL of extract/500 µL of syrup). Two-Way ANOVA: supplementation method F(2, 222) = 531.86; *p* < 0.0001, se ± 0.0106488803–0.0127888916; days of supplementation: F(4, 222) = 2707.86, *p* < 0.0001, se ± 0.0141047865–0.0211571798; supplementation method × days of supplementation F(6, 222) = 341.62, *p* < 0.0001, se ± 0.0211571798–0.0287469466. Data are presented as least squares means (LS-means), and error bars represent standard deviations (SD). Different uppercase letters indicate statistically significant differences between groups (*p* < 0.05).

### 3.8. Glucose Concentrations

Glucose concentrations increased in all groups with age until days 21/28 of life. Higher values were observed in the hemolymph of workers supplemented with Ashwagandha compared to the control group; however, they were highest in group A2 until day 21 of life.

**Figure 8:**
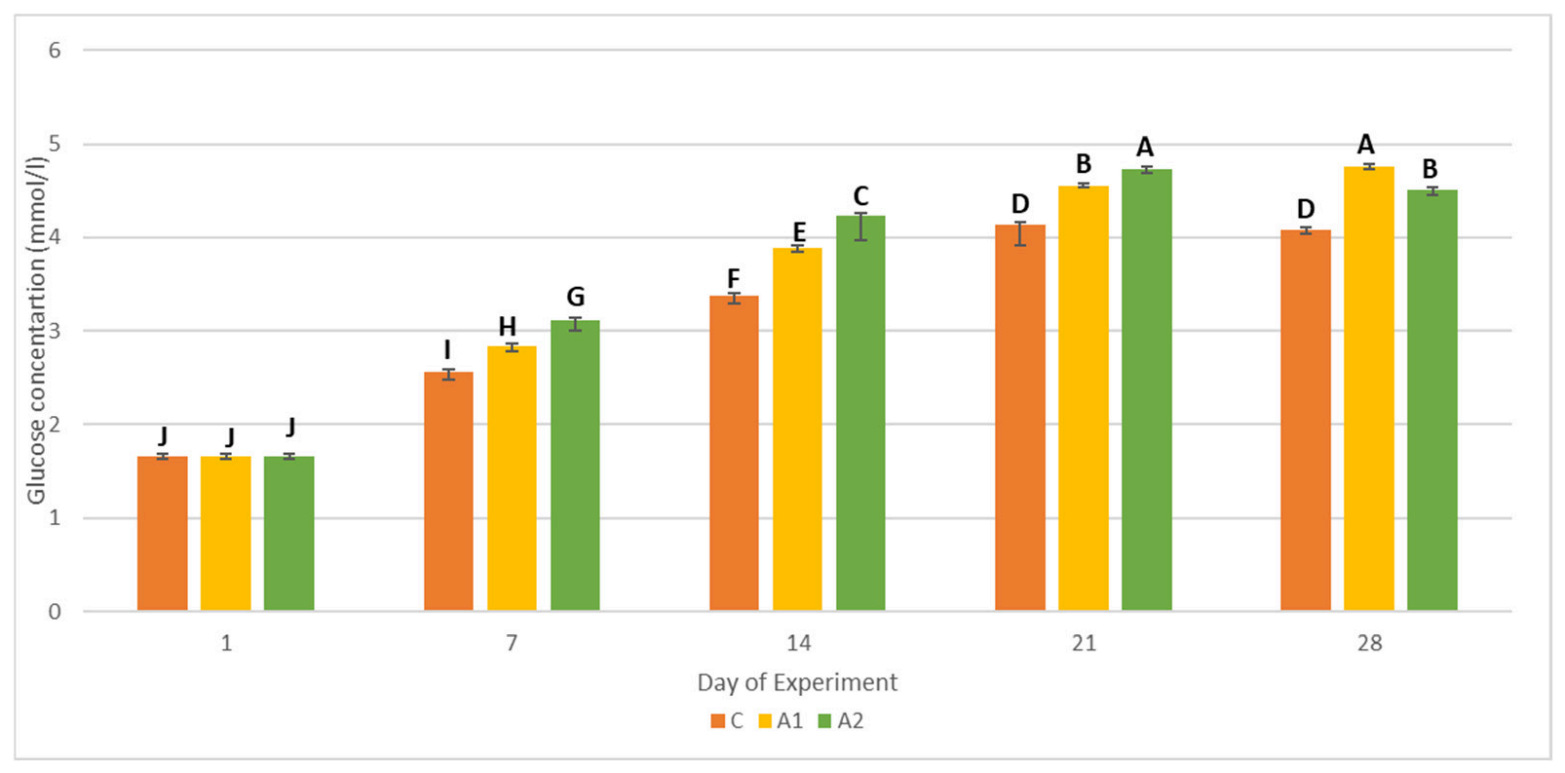
Glucose concentrations (bars represent means—$\overset{-}{x}$) in the hemolymph of workers in the control group (C)—not supplemented with Ashwagandha, and in experimental groups—supplemented with Ashwagandha (A1—10 µL of extract/500 µL of syrup, A2—20 µL of extract/500 µL of syrup). Two-Way ANOVA: supplementation method F(2, 222) = 552.79; *p* < 0.0001, se ± 0.0116878244–0.0140366231; days of supplementation: F(4, 222) = 3034.38, *p* < 0.0001, se ± 0.0154809016–0.0232213524; supplementation method × days of supplementation F(6, 222) = 27.60, *p* < 0.0001, se ± 0.0232213524–0.0315516048. Data are presented as least squares means (LS-means), and error bars represent standard deviations (SD). Different uppercase letters indicate statistically significant differences between groups (*p* < 0.05).

### 3.9. Triglyceride Concentrations

Triglyceride concentrations increased with age until days 21/28 of life in workers across all groups (in group A2, a decrease in concentration was observed on day 28). Concentrations in the hemolymph of workers supplemented with Ashwagandha were always higher than in the control group, and until day 21, they were higher in group A2 than in group A1 (on day 28, the opposite was true; higher concentrations were observed in group A1).

**Figure 9:**
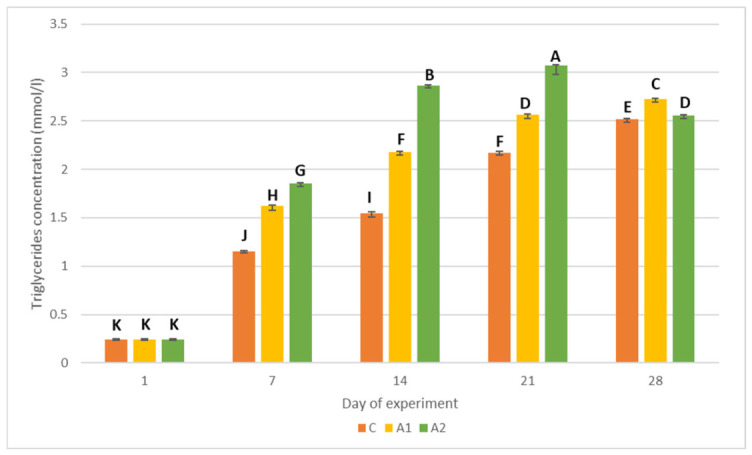
Triglyceride activities (bars represent means—$\overset{-}{x}$) in the hemolymph of workers in the control group (C)—not supplemented with Ashwagandha, and in experimental groups—supplemented with Ashwagandha (A1—10 µL of extract/500 µL of syrup, A2—20 µL of extract/500 µL of syrup). Two-Way ANOVA: supplementation method F(2, 222) = 5743.50; *p* < 0.0001, se ± 0.0041798556–0.0140366231; days of supplementation: F(4, 222) = 12,986.4, *p* < 0.0001, se ± 0.00553635399–0.00830453099; supplementation method × days of supplementation F(6, 222) = 726.99, *p* < 0.0001, se ± 0.00830453099–0.0112836357. Data are presented as least squares means (LS-means), and error bars represent standard deviations (SD). Different uppercase letters indicate statistically significant differences between groups (*p* < 0.05).

## 4. Discussion

The positive biological activity of *Withania somnifera* extract has been documented in many publications, which reported its immunomodulatory, anti-inflammatory, antioxidant, and adaptogenic properties in vertebrates. Encouraged by these scientifically confirmed effects, we used this extract for bee supplementation in the present study. Although earlier research showed toxic or growth-regulating effects of Ashwagandha in some insect species, including pest larvae and dipterans [29,30], the doses used in our experiment were much lower and selected based on previous supplementation studies in honeybees [31,32,33]. Therefore, we speculated that, as in other beneficial supplementation experiments in *Apis mellifera*, the extract would be safe for workers and could support their physiological defense mechanisms without disturbing homeostasis.

### 4.1. Total Protein Concentrations

Protein concentration in bee hemolymph is one of the biochemical markers informing about the physiological state of worker bees. Musila and Přidal (2024) [34] suggest that nutrition has a significant impact on protein concentrations in the bee body. These authors also suggest the need to know the exact age of the analyzed bees in physiological experiments, as physiological markers depend not only on nutrition but also on biological and chronological age [34]. This condition was met in our experiment. In newly emerged bees, protein concentrations are low and increase with age and pollen consumption [35,36]. In addition to pollen, substances that influence protein titers include biostimulants such as caffeine, CBD, coenzyme Q10, ascorbic acid, etc. [32,37,38]. Our research also confirmed the trend of increasing protein levels with bee age. Importantly, we demonstrated that Ashwagandha has strong stimulating potential and increases protein concentrations in the hemolymph of worker bees compared with those consuming only sugars (the control group).

The increased total protein concentration is most likely related to increased fat body activity, which plays a key role in protein synthesis, including immune proteins, in insects. This tissue is intensively bathed by hemolymph, enabling the transport of synthesized proteins throughout the honeybee’s body. Withaferin A in Ashwagandha, like curcumin and piperine, despite their different structures, exhibits the same anti-inflammatory effects. Therefore, piperine is an ingredient often combined with curcumin or Ashwagandha in supplements. This offers potential for further research in this area.

### 4.2. Proteases and Their Inhibitors

Alkaline, neutral, and acidic proteases (Figure 2, Figure 3 and Figure 4) play a crucial role in the honeybee’s immune response to pathogens. They participate in the degradation of pathogen proteins and interact with the antioxidant system, contributing to maintaining body homeostasis. Zheng et al. (2014) demonstrated that the physiological status of bees, including the activity of the proteolytic system in their hemolymph, depends on the quality and balance of the diet [39]. As Bryś et al. (2025) [40] demonstrated, the activities of proteases and their inhibitors depend on the type of pollen and were always higher in bees fed sugar enriched with pollen from entomophilous plants, not from anemophilous plants. Anemophilous plants produce pollen low in crude protein. Therefore, it can be assumed that compounds contained in the Ashwagandha plant extract stimulate the proteolytic system in the bees’ hemolymph, similarly to that in humans, and the extract itself may be a valuable supplement to a bee diet [40,41,42]. This is evidenced by the higher activity of acidic, neutral, and alkaline proteases in bees fed sugar syrup supplemented with Ashwagandha compared to the control group. A similar trend was observed for protease inhibitors (Figure 5, Figure 6 and Figure 7), except for group A2 on days 14 and 21 of the experiment. This indicates a balance between protease activities and their inhibitors, which is essential for protecting the body’s own proteins from uncontrolled degradation. When comparing two concentrations of Ashwagandha extract (10 µL or 20 µL), we demonstrated that higher additions to the sugar syrup produced a greater effect, and particularly high protease activity was observed on day 21 of the experiment. As demonstrated by Tripathi et al. (2021), compounds from the Ashwagandha extract participate in hydrogen bonding interactions with the amino acid residues Thr, Leu, Gln, Thr, and Gln, as well as in a series of hydrophobic interactions with Pro, Leu, and Met in protease inhibitors, influencing their activities [43]. Furthermore, these compounds stabilize the action of these inhibitors through polar interactions with the amino acid residues His, Asn, and Glu at specific chain positions. These responses translate into the physiological state of the body, as demonstrated by Thirunavukkarasu et al. [44] and Lee et al. [45] in studies on rats (rats administered Ashwagandha gained less weight, and their heart rate, coronary blood flow, aortic blood flow, etc., improved). Maccioni et al. [46] demonstrated that feeding TDP-43 Drosophila mutants with Ashwagandha extended their lifespan. Raut et al. (2012) demonstrated that Ashwagandha reduced total and LDL cholesterol levels, increased muscle strength, and reduced body fat [47].

In turn, Wang et al. (2021) [48] and Zhang et al. (2017) [49] reported that Ashwagandha extract exerts neuroprotective effects by activating the PI3K/Akt pathway and modulating the expression of matrix metalloproteinases (MMPs). All of these processes are directly or indirectly related to proteolysis efficiency, i.e., the activities of proteases and their inhibitors, and enable the body to maintain homeostasis. Various animal models have confirmed the anti-stress effects of Ashwagandha extracts through increased SOD and LPO activities and increased CAT and GPX activities [48,49,50]. This supports the clinical use of Ashwagandha as an anti-stress adaptogen. As previously mentioned, the antioxidant and proteolytic systems in bees are strongly interconnected, so it can be assumed that the effects observed in this study will influence antioxidant activities. This opens up further research directions.

Our results are analogous to those examining the effects of various stimulants on the proteolytic system in bee hemolymph. Examples include coenzyme Q10, caffeine, curcumin, CBD, ascorbic acid, and even fish protein hydrolysate. All of them stimulated the bee organism to increase protein synthesis and the activities of proteases and their inhibitors. Furthermore, some of them demonstrated protective effects, for example, against *Vairimorpha*/*Nosema* spp. [37,51,52,53]. It remains to be seen whether Ashwagandha extract will also have this effect, which is worth exploring in the future.

### 4.3. Concentrations of Biochemical Markers

A gradual increase in glucose and triglyceride concentrations was observed throughout the life of the bees (Figure 8 and Figure 9). Increased glucose levels indicate continued proper food intake and its availability as an energy substrate for metabolic processes. At approximately 21 days of age, honeybees enter the foraging phase and begin intensive flight, which requires significant energy. From day 1 to day 21 of life, energy substrates are stored primarily in the fat body.

Because the bees in this experiment were kept in cages and did not forage, stored energy substrates could have accumulated further. Higher glucose and triglyceride concentrations were observed in the groups supplemented with Ashwagandha extract compared with the control group. Therefore, it can be concluded that simultaneous immune stimulation and preservation of the protease–inhibitor balance may promote the accumulation of energetic reserves in the honeybee body, which can subsequently be used to support defense reactions and metabolic demands. Moreover, the increase in glucose and triglycerides on days 21 and 28 coincided with elevated acid protease activity, which is consistent with the role of proteolytic and energetic remodeling during normal worker development. In honeybees, age-related changes in energy metabolism are closely linked to the efficiency of the Krebs cycle and respiratory chain, and in naturally aging workers, the highest values of metabolic components are usually observed around day 28, particularly in the fat body of tergite 3. Tergite 3 has previously been identified as the main site of energy storage, with the highest levels of glucose, glycogen, and triglycerides, which supports its role as a key metabolic reservoir in the worker’s body [54]. Thus, the observed changes suggest that Ashwagandha extract may help maintain normal metabolic development in honeybees exposed to this treatment.

The results demonstrate significant similarity to previously observed changes in honeybee immune systems following CBD supplementation. In the study by Skowronek et al., both the proteolytic system and non-enzymatic markers demonstrated similar activity and concentration in the hemolymph. Although CBD and withaferin A belong to distinct chemical groups and differ in structure, in mammals, they interact with the common NF-κB signaling pathway, leading to anti-inflammatory effects, reduced oxidative stress, and normalization of the HPA axis.

The honeybee lacks the classic NF-κB pathway, but possesses its evolutionarily homologous, simplified counterparts—the Toll and Imd (immune deficiency) pathways. Both of these pathways lead to the activation of the transcription factors Dorsal and Relish, which are functional equivalents of the mammalian NF-κB/Rel family. Considering the above similarities and the similar results obtained after CBD supplementation, it can be assumed that withaferin A—the main component of Ashwagandha—affects the honeybee immune system in a functionally similar manner, albeit via a different molecular pathway. Therefore, further molecular studies on the mechanism of action of these extracts in the honeybee seem fully justified.

### 4.4. Comparison of Supplementation Results with Results of Exposure to Negative Factors

Nowadays, it would be impossible to avoid using pesticides in agriculture. Although their positive effect on crops is invaluable, they have a highly negative impact on pollinating insects, including honeybees. Imidacloprid, a representative of neonicotinoids, affects honeybees not only physiologically, but also at the behavioral and genetic levels. It increases the concentration of toxic elements in their bodies, causes motor problems (e.g., spatial orientation, mobility, and return to the hive disorders) and modulates the expression of genes responsible for the biosynthesis of antioxidant enzymes [55,56,57,58]. In addition, lower proteolytic activity of proteins in the organism of bees was demonstrated, indicating the destructive effect of pesticides compared to the action of Ashwagandha extracts [59].

Not only pesticides, but also electromagnetic fields generated by human activity have a negative impact on the proteolytic system of honeybees. As Migdał et al. (2021) [60] noted, their intensity in the environment is constantly increasing due to the growing number of electrical devices. Even a field with a frequency of 50 Hz affects the activity of proteases in honeybees. Acid proteases exposed to this field show significantly higher activity than in the control group at each tested intensity (5.0, 11.5, 23.0, 34.5 kV/m). In contrast, alkaline proteases exhibit only slightly higher activity than the control at intensities of 11.5 and 34.5 kV/m. Although these effects do not contradict the effects of Ashwagandha extract, it is worth looking at the key differences in the research results. The electromagnetic field was applied in these studies on 1-day-old bees for a period of 1 day, while Ashwagandha extract was used throughout their entire lifespan. Furthermore, studies involving the effects of electromagnetic fields did not include protease inhibitor studies [60]. Considering the proteolytic balance of Ashwagandha extract, its positive effect on honeybees can be demonstrated in comparison to electromagnetic fields.

### 4.5. Lepidoptera Versus Apis mellifera (Hymenoptera)

It is worth noting that *Withania somnifera* (Ashwagandha), specifically its root powder and leaf extracts, is frequently used as an insecticide against crop pest larvae. It negatively impacts the metamorphosis and molting of Lepidoptera caterpillars (e.g., *Spodoptera litura*) by interfering with ecdysone and juvenile hormones, leading to impaired larval, pupal, and adult development, reduced survival, and inhibition of digestive/detoxification enzymes [28]. Similar effects occur in Diptera, including Calliphoridae (*Chrysomya megacephala*): prolonged larval-pupal and pupal-imaginal ecdyses, larval mortality, abnormal pupariation, and adult deformities. Furthermore, studies mentioned above highlight the importance of dosage for honeybees as an insect, as well as the crucial role of honeybee social immunity in mitigating these risks. Gaur et al. [28,29] also assessed immunology post-eclosion (after metamorphosis), suggesting that honeybee social mechanisms may counteract Ashwagandha’s negative larval effects observed in Lepidoptera [29,30]. This provides a promising starting point for testing this hypothesis in future apiary experiments.

## 5. Conclusions

The correlation between the results regarding the proteolytic system and non-enzymatic markers suggests that Ashwagandha acts as a stimulant of the honeybee’s immune system while maintaining its homeostasis. Continued research, particularly in the area of antioxidant activity and apiary tests, may enable the assessment of the potential use of Ashwagandha extract in beekeeping practices as a means of supporting the mobilization of honeybee immunity.

## Data Availability

Data contained within the article. At a justified request of the interested party, they may be made available by the corresponding author.
